# Supplementary figures and images for: Annulus Fibrosus Injury Induces Acute Neuroinflammation and Chronic Glial Response in Dorsal Root Ganglion and Spinal Cord—An In Vivo Rat Discogenic Pain Model
Source: Int J Mol Sci. 2024 Feb 1;25(3):1762. doi: 10.3390/ijms25031762 (PMC10855200; doi:10.3390/ijms25031762)

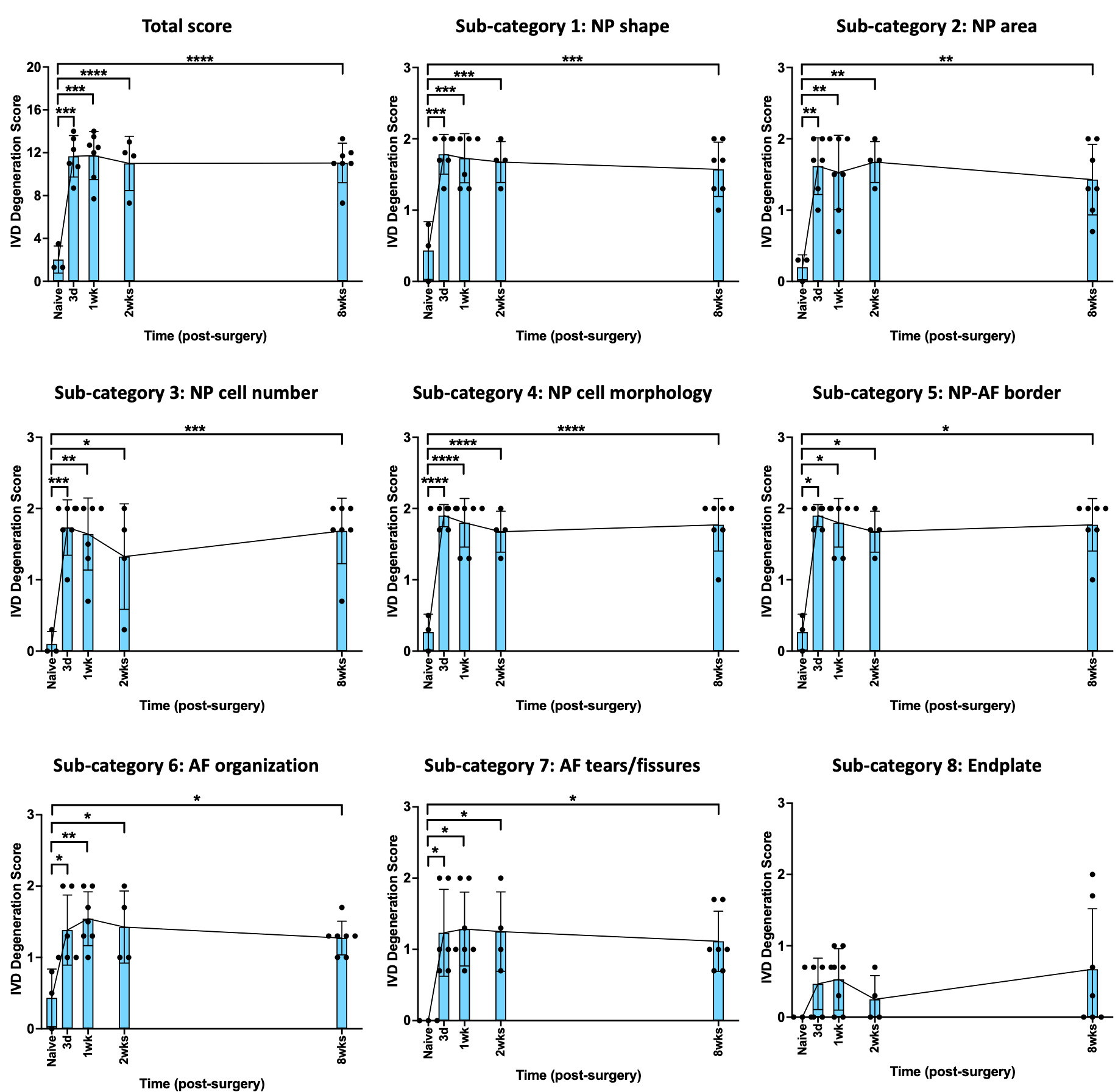

Supplement: Supplementary file 1 [file ijms-25-01762-s001.zip › FigureS1.tiff]

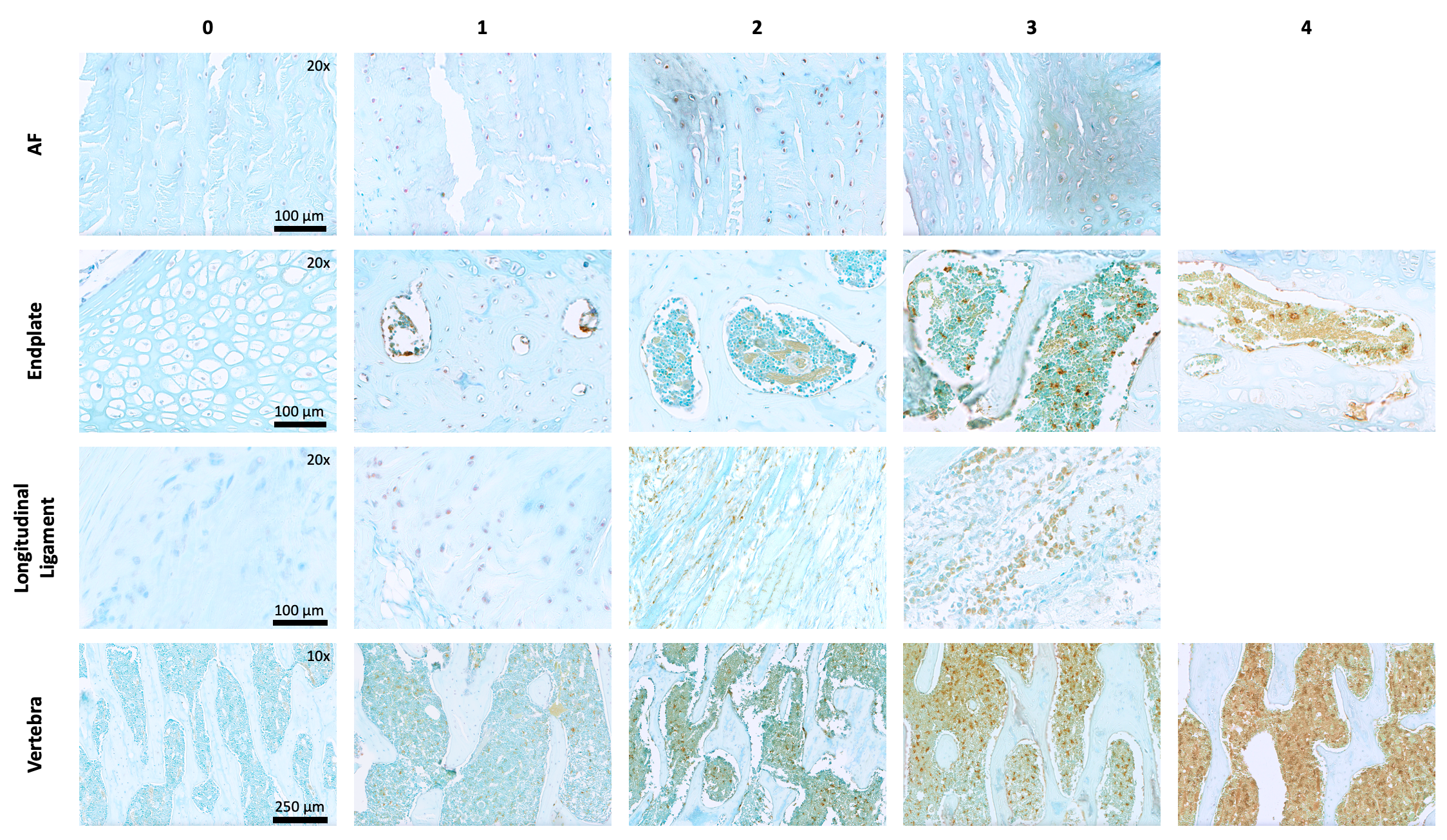

Supplement: Supplementary file 1 [file ijms-25-01762-s001.zip › FigureS2.tiff]

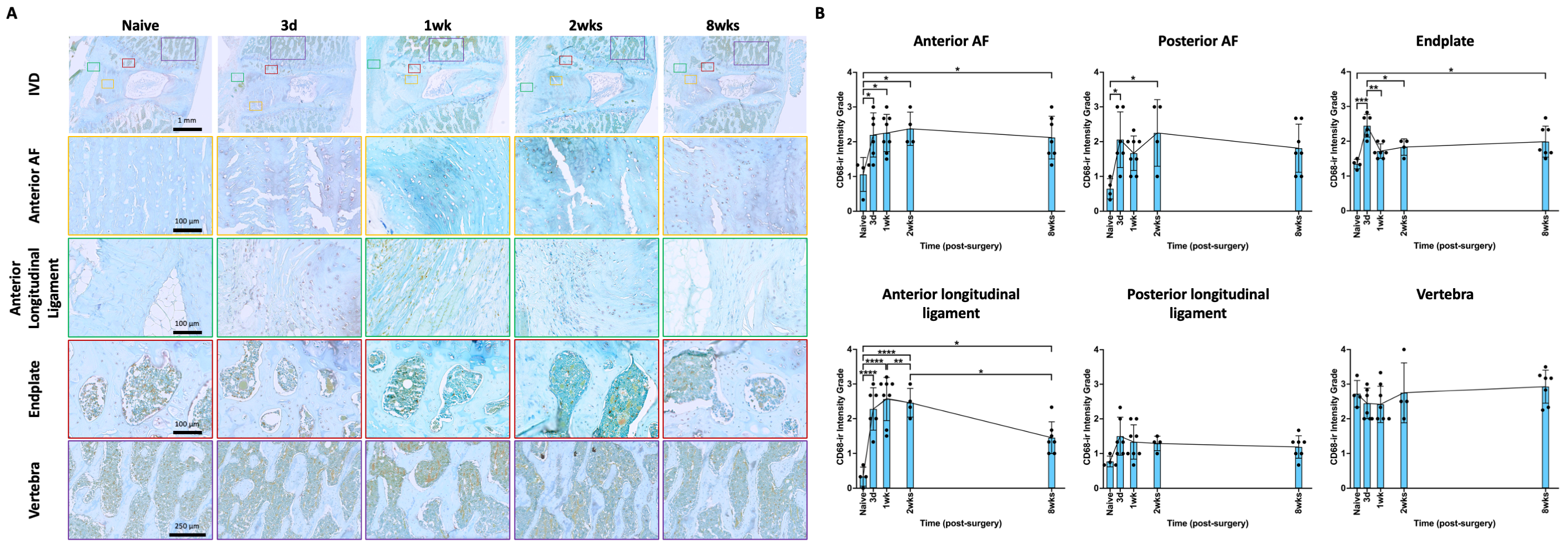

Supplement: Supplementary file 1 [file ijms-25-01762-s001.zip › FigureS3.tiff]
